# Hyperglycaemia in pregnancy: knowledge and correlates amongst antenatal care providers in healthcare facilities in Jos, Nigeria

**DOI:** 10.4314/ahs.v23i1.40

**Published:** 2023-03

**Authors:** Lucius Imoh, Abdulazis Longwap, Nicholas Yersib, Dungrit Gowok, Zainab Muhammad, Joy Imoh, Mathilda Banwat

**Affiliations:** 1 University of Jos, Department of Chemical Pathology; 2 University of Jos, Department of Geography and Planning; 3 University of Jos, Department of Community Medicine

**Keywords:** Hyperglycaemia in Pregnancy, gestational diabetes mellitus, knowledge of GDM, guidelines for GDM

## Abstract

**Background:**

Antenatal healthcare providers' (AHPs) knowledge about hyperglycaemia in pregnancy (HIP) and its screening best practices affect the management of affected pregnant women. We assessed the knowledge of HIP and associated factors amongst first line AHPs.

**Methods:**

This descriptive cross-sectional study involved 188 Doctors, Nurses and Community Health providers directly involved in providing antenatal care at all levels of health care in Jos, Plateau State, Nigeria, selected through total sampling technique.

**Result:**

A total of 103 AHPs (54.8%) were females. The mean knowledge score (SD) score was 17.0+/-5.5 (out of 30). Only 93 (49.5%) had a good knowledge of HIP (Knowledge score ≥18). Only 88 (46.8%) could correctly identify 75g OGTT or 100g OGTT as diagnostic tests for GDM. Gender, category of hospital, level of care of the institution and job designation were significantly associated with knowledge of HIP after bivariate analysis (p < 0.05). After multivariate analysis using logistic regression analysis, only the category of institution and job designation were independently associated with knowledge of HIP.

**Conclusion:**

The general level of knowledge of HIP among AHPs is average but awareness of testing and management guidelines is very poor hence the need for regular updates for health professionals.

## Introduction

Hyperglycemia in pregnancy (HIP) is one of the most common medical conditions complicating pregnancy.^1^ HIP, manifesting as gestational or pre-gestational (Overt) diabetes mellitus is a leading cause of adverse pregnancy outcomes globally.^2^ Hyperglycaemia and other metabolic derangements due to and Overt Diabetes Mellitus in pregnancy (ODM) are associated with an increased risk of maternal and foetal complications.^1^,^2^ GDM is a harbinger for future DM and cardiovascular disease in women. Women diagnosed with GDM have an increased risk of progressing to DM post-delivery.^3^,^4^ Moreover, metabolic conditioning of the foetal cells, due to persistent hyperglycaemic milieu in-utero, is thought to programme the foetus for future obesity and DM; thus, perpetrating a vicious circle of NCDs in successive generations.^5^

Considering the high fertility rate and the sheer large number of women within the reproductive age bracket in Nigeria,^6^ screening for GDM and Overt DM in pregnancy could be considered an important public health measure for improving maternal and child health. Moreover, screening for HIP offers a window of opportunity for preventing intergenerational transmission of NCDs.^5^

Antenatal care providers are crucial in any screening programme for HIP and therefore the overall health of pregnant women. First-line health care providers, particularly nurses and midwives are usually the first point of contact for pregnant women. Pregnant women obtain a considerable amount of information regarding their health and that of their unborn child from health workers who provide antenatal care. Indeed, hyperglycaemia in pregnancy is one of the important information that AHPs are expected to provide to expectant mothers during antenatal clinic sessions. In a survey in Rivers State, Nigeria, health workers were a major source of information on GDM according to 34.6% of the respondents.^7^ This data suggest that AHPs have a key role to bridge the knowledge gap on HIP.

Pregnant women also rely on Antenatal care providers to determine if they will be screened, when and how they will be screened for HIP. Referral patterns and screening practices adopted by antenatal health care providers even within a single tertiary hospital setting have been shown to vary considerably.^8^ The decisions regarding screening for HIP are influenced by several factors including the knowledge of screening modalities for HIP by antenatal care provider as well as available resources in healthcare facilities.

The knowledge of these health workers about HIP and the best practices for screening for HIP will also impact their management of pregnant women with HIP. Unfortunately, there is paucity of data on the subject among antenatal caregivers in Nigeria. Most studies on GDM and DM in pregnancies have focused on women assessing care or health workers in tertiary and academic health care centres. This study will assess the knowledge of HIP amongst first line antenatal healthcare providers (AHPs) in all tiers of health care facilities. The result from this study will highlight their potentials as well as limitations in the screening and management of HIP.

## Materials and Methods

### Study Area

This cross-sectional study was conducted amongst AHPs of different cadres at all levels of health care - primary, secondary and tertiary health care centres in Jos North Local Government Area. Plateau state is located in the North-central geo-political zone of Nigeria. Jos North is one of the Local Government Area of Jos, the capital city of Plateau state is cosmopolitan, inhabiting people from all tribes in Nigeria.

These study participants included Doctors, Nurses and Community Health providers (Front-line staff) directly involved in providing antenatal care in primary, secondary and tertiary health care centres in Jos who gave consent to participate in the study. AHPs on leave or unavoidably absent during the period of the study were excluded from the study.

The sample size was determined by the formula for cross-sectional study and finite population correction.^9^,^10^ Using an assumed prevalence of screening for HIP of 50% and population size of AHPs in Jos North Local Government Area of 140. A value of 10% of the minimum sample size was added to the study to account for non-response and incomplete data bringing the total sample size to 115 AHPs.

### Sampling Technique

A total sampling of all the 69 health facilities and all eligible AHPs in the health facilities were targeted for the study. All AHPs that meet the inclusion criteria were included in the study and were provided with a semi-structured questionnaire to complete and return.

### Data Collection Instrument

Data were collected using a pretested self-administered structured questionnaire consisting of three parts (part A [socio-demographics], B [Basic Knowledge of HIP], C [ Knowledge of screening guidelines for HIP]. The questionnaire was developed following an extensive review of the available literature and was written in the English Language given the high level of English fluency among the target participants. The participants were allowed to select all options that apply to a given question and the freedom to select “Not sure” if they were uncertain of the response. A composite knowledge score was computed from the responses to nine questions with 30 correct answers assessing basic knowledge HIP and the recommended screening practices for HIP. The knowledge score of the AHPs was classified as good if they score ≥ 18 (≥60%) and poor knowledge if they scored ≤ 59%.

### Procedure for Data Collection

At each selected health facility, each eligible respondent was given a detailed explanation of the research by the researcher or trained research assistants. After obtaining informed consent the questionnaire was distributed to the participants who self administered them independently and returned to the researcher on completion. The researcher and trained research assistants served as a supervisor to ensure data quality by checking the completeness of answers.

### Data Analysis

Data were cleaned and entered in Microsoft Excel® version 2.0 and exported to Statistical Product and Service Solutions (SPSS) version 23.0 (IBM Corp. 2015, Armonk, NY) software for statistical analysis. Descriptive statistics were presented as mean values +/- standard deviation (SD) or medians with interquartile ranges (IQRs) for none normal continuous variables, and proportions (as percentages) for categorical variables. Tables and graphical representations were used to summarise the data. Statistical associations of dependent and independent variables were assessed using Chi-square tests or Yate's correction test for continuity where Chi-square test will not be appropriate. Multivariate analysis using Logistic regression was used to assess determinants of knowledge and practices of HIP screening. All tests were 2-tailed, a 95% confidence interval was used and P-values of less than 0.05 (P <0.05) was considered statistically significant.

### Ethical Consideration

This study was carried out after due approval from the Human Research Ethical Committee (HREC) of the Jos University Teaching Hospital (JUTH). Written permission was obtained from the Plateau State Ministry of Health. Appropriate permission was obtained from the relevant authorities of the health facilities. Written informed consent was obtained from all participants after due explanation of the research work and procedure. Anonymity and confidentiality of the information obtained from the participants in this study was assured and maintained.

## Result

A total of 250 questionnaires were distributed to AHPs in 60 health facilities of which 193 were returned giving a response rate of 77.2%. However, only 188 with completed questionnaires were analysed.

### Socio-demographics of Respondents

In Table 85 (45.2%) of the respondents were males and 103 (54.8%) were females. The ages of the participants ranged from 20 years to 60 years with a mean of 35.7 +/-8.5 years. Most (51.1%) of the respondents worked in private health facilities and 42% provided primary health care. Sixty-seven (35.6%) of the participants were doctors and the majority of them described their role as Medical Officer (28.4%). Obstetrics and Gynaecology (48.5%) was the most common specialty among doctors with clinical specialties. The summary of the socio-demographic characteristics of the study participants is also shown.

In table 2, out of a Maximum knowledge score of 30, the mean +/- SD score was 17.0 +/- 5.5. Only 93 (49.5%) had a good knowledge of HIP. One hundred and thirty-eight (73.3%) of the respondents correctly defined HIP, however only 78 (41.5%) and 107 (56.9%) could correctly define Diabetes in pregnancy and GDM respectively. Eighty-two (43.6%), and 33 (17.6%) could correctly identify all the risk factors for GDM and the consequences of GDM respectively. Mean +/- SD knowledge score of risk factors for GDM was 6.1 +/- 2.5 from a maximum of 8 while for consequences of GDM the score was 6.8 +/- 3.1 from a maximum of 11. Only 88 (46.8%) could correctly identify 75g OGTT or 100g OGTT as diagnostic tests for GDM.

**Table 2:** Knowledge of Hyperglycaemia in Pregnancy (HIP)

Variable	Frequency	Percentage
Correct definition of DM in pregnancy	78	41.5
Correct definition of GDM	107	56.9
Correct definition of HIP	138	73.3
Correct identification of GDM risk factors	82	43.6
Correct identification of consequences of GDM	33	17.6
Correct identification of diagnostic test for GDM	88	46.8
% Knowledge Score ≥ 60 (Good Knowledge)	93	49.5
% Knowledge Score ≤ 59 (Poor Knowledge)	95	50.5
knowledge score of risk factors for GDM (Mean +/-SD)		6.1 +/-2.5
knowledge score of consequences of GDM (Mean +/-SD)		6.8 +/-3.1
**Aware of Nigerian Guideline (n=128)**		
Yes	37	28.9
No	91	71.1
**Correctly Identified any Screening Guideline (n=128)**		
Yes	16	12.5
No	112	87.5
**Identified Screening Guidelines (n=16)**		
WHO	13	10.2
ADA	3	2.3
ACOG	1	0.8
NICE	1	0.8
HAPO	1	0.8

Only 37 (28.9%) from a subset of participants (n=128) who screened for GDM were aware of a Nigerian guideline for screening and management of GDM and only 16 (12.5%) of these could correctly identify at least one guideline for screening and management of GDM. World Health Organization (WHO) guideline (10.2%) was the most commonly identified by them. Only 0.8% each correctly identified American Diabetes Association (ADA), National Institute for Health and Care Excellence (NICE) and Hyperglycemia and Adverse Pregnancy Outcome (HAPO) study guidelines.

In Table 3, Gender, Category of hospital, level of care of the institution and job designation were significantly associated with knowledge of HIP after bivariate analysis (p < 0.05). Proportionally, males compared to females were significantly more knowledgeable about HIP. The category of hospital was significantly associated with knowledge of HIP with Antenatal care providers in Faith-based and government/public hospitals showing more knowledge of HIP than their private hospital counter-part. Similarly, antenatal care providers in tertiary care facilities and Doctors in particular were more knowledgeable about HIP. The age, cadre of doctor, specialty and additional qualification were not significantly associated with knowledge of HIP. After multivariate analysis using logistic regression analysis, only category of institution and Job designation were independently associated with knowledge of HIP.

**Table 3:** Bivariate and Multivariate analysis of factors associated with knowledge of hyperglycaemia in pregnancy

Variable	Good Knowledge (%)	Poor Knowledge (%)	P-value	Adjusted P-value
**Sex**			**0.001**	0.864
Male	53 (62.4)	32 (37.6)		
Female	40 (38.8)	63 (61.2)		
**Age Group**			0.475	
20–29	19 (41.3)	27 (58.7)		
30–39	44 (51.8)	41 (48.2)		
40–49	20 (48.8)	21 (51.2)		
50–60	10 (62.5)	6 (37.5)		
**Institution Category**			**0.030**	**0.030**
Faith-Based	11 (78.6)	3 (21,4)		
Government/Public	46 (46.9)	52 (53.1)		
Private	30 (39.5)	46 (60.5)		
**Level Of Institution**			**0.001**	0.424
Primary	28 (35.4)	51 (64.6)		
Secondary	25 (51.0)	24 (49.0)		
Tertiary	40 (66.7)	20 (33.3)		
**Job Designation**			**P<0.001**	**<0.001**
Other	3 (27.3)	8 (72.3)		
CHO	6 (54.5)	5 (45.5)		
CHEW	14 (35.0)	26 (65.0)		
Nurse/Midwife	14 (23.7)	45 (76.3)		
Doctor	56 (83.6)	11 (16.4)		
**Cadre Of Doctor**			0.531	
House Officer	7 (70.0)	3 (30.0)		
Medical Officer	16 (84.2)	3 (15.8)		
Registrar	5 (83.3)	1 (16.7)		
Senior Registrar	9 (90.0)	1 (10.0)		
Consultant	16 (94.1)	1 (5.9)		
**Doctors Specialty**			0.735	
Family Medicine	8 (88.9)	1 (11.1)		
Obs & Gynae	15 (93.8)	1 (6.3)		

## Discussion

This study assessed the level of knowledge about basic aspects of HIP among antenatal care providers. In general, the level of knowledge of HIP was about average with just under fifty percent of participants demonstrating an overall good knowledge of HIP. Previous studies have demonstrated a poor level of knowledge of GDM and its management among health care providers. 11–13 Although a good number of the AHPs could correctly define HIP, majority of them had difficulty classifying the different types of Hyperglycaemia in pregnancy. Whereas in this study, only 57% of the AHPs could correctly describe GDM, almost 90% of AHPs surveyed in a similar study in Morocco correctly described GDM.^14^ The subject of HIP is very dynamic with several changes in guidelines in recent years. In the absence of regular updates, a recent global action to classify HIP based on the degree of hyperglycaemia resulting in separation of mild from severe hyperglycaemia may have been responsible for AHPs having difficulty classifying the types of HIP. The greater knowledge gap in this study was identifying the consequences of GDM and the correct diagnostic test for GDM. A previous study had shown that health workers either underestimated or were unsure of the future risk of hyperglycaemia in pregnancy.^15^The variability in the screening or diagnostic tests among providers of antenatal care has also been highlighted.^16^–^18^ This poor knowledge of HIP and inconsistent classification of HIP among AHPs may impact the management and referral of this condition within and between centres. Also, the poor knowledge about HIP may have implications for adequate counselling of pregnant women. This is because the knowledge about HIP in the general Nigerian population has been shown to be poor.^7^ Antenatal care providers who are expected to bridge this knowledge gap are rendered ineffective if they are not up to date with requisite knowledge of HIP.

When disaggregated among categories of AHPs, doctors expectedly were more grounded in knowledge about HIP compared to other health workers. This was in keeping with the finding in a study among health workers in Morroco.^14^ Also, Adeleke et al, reported that doctors overall had better knowledge of GDM than nurses.^12^ This is likely to reflect their differentially higher training on issues regarding HIP. Also, AHPs working in Faith-based health centres were more likely to have good knowledge of HIP compared to their counterpart in government or public centres. An explanation for this is that some public health antenatal centres in this study were manned by CHOs and CHEW with low-level knowledge of HIP. Knowledge of HIP in this study was not associated with the cadre of doctors or specialty practised by the doctors. This may suggest a poor update on the current trends on HIP among AHPs. A survey on GDM among doctors also found that knowledge was independent of specialty, seniority, academia, years in practice or country trained.^19^

Among AHPs who screen for GDM, more than 70% are not aware of a Nigerian guideline for screening and management of GDM and only about 10% could correctly identify any screening and management guideline, all of these were doctors. The WHO guideline was the most commonly identified. In a survey among doctors in Bangladesh, more than half (53.4%) used the OGTT-criteria, suggested by the American Diabetes Association (ADA), and 9.5% used the World WHO criteria while 31.3% used fasting plasma glucose (FPG) and plasma glucose 2-hours after breakfast (PG-2HABF).^20^ Our findings suggest a lack of knowledge of standard practices for screening for HIP. It also buttresses earlier reports of lack of coordination or local consensus as far as screening and management of GDM are concerned.^19^,^21^ The implication is that respondents carry out screening practices but are not aware of the authority recommending such practices and therefore the basis for such practice. This negates the principle of evidence-based medicine with implications for the standard of care for pregnant women.

There are several guidelines and recommendations for screening for GDM, many issued by international Diabetes and Obstetrics Associations. The widely recognized ones include:21 The World Health Organization; WHO (revised in 2013), America Diabetes Association (ADA), American College of Obstetricians and Gynecologists (ACOG), National Institute for Health and Care Excellence (NICE) and International Federation of Gynaecology and Obstetrics (FIGO)^21^. These guidelines have considerable overlaps, however, there are major areas of discrepancies. It is generally advocated that countries should develop local guidelines to direct screening and the management practices for HIP in line with local realities.^5^,^21^,^22^ In Nigeria, attempt to provide guiding principles for screening and managing HIP have been provided by organizations such as the Diabetic Association of Nigerian (DAN.^23^ The Society of Gynaecology and Obstetrics of Nigeria (SOGON) has put out guidelines on management of some obstetrics problems such as Post-partum Haemorrhage, Pre-eclampsia and Eclampsia and cervical cancer prevention on their website. However, they have not published a statement or guideline on screening and management of HIP.^24^

The result from this study suggests that AHPs are largely unaware of any Nigerian guidelines. This may be a result of poor circulation and publicity of the DAN guideline. The guidelines are likely beyond the reach of AHPs, especially in primary or secondary care settings. Unless a concerted effort is made to step down the information contained in these guidelines to AHPs, this situation may likely remain the same. Another plausible reason for poor awareness of Nigerian guidelines is that the Nigerian guidelines appear to be more or less wholesale adoptions of the internationally recognized guidelines with little or no modifications to reflect local peculiarities. The Nigerian guidelines will be more relevant for AHPs if they can provide strategies for screening and managing HIP that take into cognizance the peculiarities and challenges in healthcare delivery in Nigeria for instance relating to the availability of manpower and equipment.

Communication of important guidelines to the health workers is crucial. Unfortunately, a study suggests that the majority of AHP lack training on HIP and many are not updated regarding current screening practice or HIP. Only 56.8% of doctors and 23.3% of nurses reported receiving pre-service training on GDM but only 10% of the respondent have had on-the-job training on HIP.^13^ It therefore crucial that AHPs are up to date on current trends regarding HIP.

This study may have had some limitations. As this is a cross-sectional study, it may be difficult to establish a temporal relationship between knowledge and practice on HIP. Also, the response of the AHPs on the issues concerning HIP knowledge and screening practices may be influenced by social desirability bias. The level of knowledge of HIP may affect the understanding and therefore responses of the respondents in areas where adequate knowledge is requisite for a comprehensive response. However, an effort was made to reduce ambiguity in designing the questions, and through pre-testing. Standard abbreviations were initially fully annotated.

## Conclusion and recommendation

This study has provided rare data on the level of knowledge of HIP among AHPs in health facilities in Jos North and describe the screening practices for GDM employed by the AHPs. Although doctors show more knowledge about HIP compared to other categories of antenatal care providers, the general level of knowledge is below average. The classification of HIP is not up to date and awareness of guidelines including local (Nigeria) guidelines is very poor.

In light of these, there should be deliberate attempts by regulators of medical education to increase the basic knowledge of HIP including, screening, diagnosis and management practices among health workers at all cadres by ensuring that adequate content of this subject in the various curricular of training for these professionals. HIP is a rapidly evolving area of medicine and regular updates should be provided to AHPs through established fora for medical updates to health workers e.g., through Continuing Medical Education (CMEs) platforms and update courses. Training for AHPs on screening practices and risk assessment for HIP in primary health care settings is particularly important. This can be provided by Specialist obstetricians, Endocrinologists and laboratorians. This will provide the much needed up to date screening practice for HIP. It is commendable that in the last decade gains have been made to harmonious international guidelines. It is therefore crucial that stakeholders in Nigeria work at developing consensus to address the peculiarities of screening in the Nigerian context. The Federal Ministry of Health in Nigeria, working together with diabetic associations, obstetrics associations and other professional associations should take the lead to harmonize screening practice and management practice for HIP. There is a need for proper circulation of screening and management guidelines for GDM in an easily understood format. Lower cadre AHPs should be carried along in producing useful guidelines suitable for primary and secondary settings. This could be provided by the National and State Ministry of Health as well as Diabetes and Obstetrics Societies in Nigeria. This would provide the needed education and consensus guiding principles for HIP screening and management by AHPs.

## Figures and Tables

**Table 1:** General Characteristics of Study Participants

Variable	Frequency	Percentage
**Age**		
Mean (SD)	35.7 (8.5)	
**Sex**		
Male	85	45.2
Female	103	54.8
**Institution Category**		
Faith-Based	14	7.5
Private	96	51.1
Government/Public	78	40.4
**Level Of Institution**		
Primary	79	42.0
Secondary	49	26.1
Tertiary	61	31.9
**Job Designation**		
Doctor	67	35.6
Nurse/Midwife	59	31.4
CHO*	11	5.9
CHEW*	40	21.3
Non-specified	11	5.8
**Cadre of Doctors**	n=67	
House Officer	10	14.9
Medical Officer	19	28.4
Registrar	6	9.0
Senior Registrar	10	14.9
Consultant	17	25.4
Non-specified	5	7.5
**Doctors Specialty**	n=25	
Family Medicine	9	36.0
Obstetrics & Gynaecology	16	64.0

* CHO = Community Health Officer, CHEW = Community Health Extension Workers

## References

[1] Farrar D (2016). Hyperglycemia in pregnancy: prevalence, impact, and management challenges. Int J Womens Health.

[2] American Diabetes Association (2017). Classification and Diagnosis of Diabetes. Diabetes Care.

[3] Debjyoti K, Jai SB (2010). Diabetes mellitus in pregnancy. JIMSA.

[4] Burlina S, Dalfrà MG, Chilelli NC, Lapolla A (2016). Gestational Diabetes Mellitus and Future Cardiovascular Risk: An Update. Int J Endocrinol.

[5] Hod M, Kapur A, Sacks DA, Hadar E, Agarwal M, Di Renzo GC (2015). The International Federation of Gynecology and Obstetrics (FIGO) Initiative on gestational diabetes mellitus: A pragmatic guide for diagnosis, management, and care. Int J Gynecol Obstet.

[6] Adebowale AS (2019). Ethnic disparities in fertility and its determinants in Nigeria. Fertil Res and Pract.

[7] Ogu RN, Maduka O, Agala V, Alamina F, Adebiyi O, Edewor U (2020). Gestational Diabetes Mellitus Knowledge Among Women of Reproductive Age in Southern Nigeria: Implications for Diabetes Education. Int Q Community Health Educ.

[8] Imoh LC, Ogunkeye OO, Daru PH, Amadu NO, Abu A, Asorose SA (2015). Appraisal of timing for oral glucose tolerance testing in relation to risk factors for gestational diabetes mellitus in pregnant women in a Nigerian Teaching Hospital. Niger J Clin Pract.

[9] Kasiulevicius V, Sapoka V, Filipaviciute R (2006). Sample size calculation in epidemiological studies. Gerontology.

[10] Daniel WW (1999). Biostatistics: A Foundation for Analysis in the Health Sciences.

[11] Patel S, Vyas S (2018). Evaluation of Training Program about Awareness of Gestational Diabetes Mellitus (GDM) among Health Care Workers of Ahmedabad Municipal Corporation. Natl J Community Medicine.

[12] Adeleke NA, Olowookere SA, Farinloye EO, Abiodun OM, Alebiosu CO (2014). Management of gestational diabetes mellitus at secondary health care level: a survey of ante-natal care givers' knowledge, attitude and practice.

[13] Appajigol JS, Bellary S (2015). Knowledge and practices of rural family physicians and obstetricians towards gestational diabetes mellitus. Int J Community Med Public Health.

[14] Utz B, Assarag B, Essolbi A, Barkat A, Delamou A, De Brouwere V (2017). Knowledge and practice related to gestational diabetes among primary health care providers in Morocco: Potential for a defragmentation of care?. Prim Care Diabetes.

[15] Hanson P, Huda MS, Hitman GA, Thangaratinam S (2015). Postpartum care of women with gestational diabetes: survey of healthcare professionals. Eur J Obstet Gynecol Reprod Biol.

[16] Mukuve A, Noorani M, Sendagire I (2020). Magnitude of screening for gestational diabetes mellitus in an urban setting in Tanzania; a cross-sectional analytic study. BMC Pregnancy Childbirth.

[17] Agarwal MM (2018). Consensus in Gestational Diabetes Mellitus: Looking for the Holy Grail. J Clin Med.

[18] Utz B, De Brouwere V (2016). “Why screen if we cannot follow-up and manage?” Challenges for gestational diabetes screening and management in low and lower-middle income countries: Results of a cross-sectional survey. BMC Pregnancy Childbirth.

[19] Agarwal MM, Shah SM, Kaabi J Al, Saquib S, Othman Y (2014). Gestational diabetes mellitus: Confusion among medical doctors caused by multiple international criteria. J Obstet Gynaecol Res.

[20] Selim S, Kazal RK, Kamrul-Hasan ABM (2020). Knowledge and Practice of Physicians to Hyperglycemia in Pregnancy: A Nationwide Survey in Bangladesh. Int J Diab Endocrinol.

[21] Utz B, Kolsteren P, De Brouwere V (2015). Screening for gestational diabetes mellitus: Are guidelines from high-income settings applicable to poorer countries?. Clin Diabetes.

[22] Adam S, Rheeder P (2017). Selective Screening Strategies for Gestational Diabetes: A Prospective Cohort Observational Study. J Diabetes Res.

[23] Diabetes Association of Nigeria (2013). Clinical Practice Guidelines for Diabetes Management in Nigeria.

[24] Society of Gynaecology and Obstetrics of Nigeria (SOGON).

